# FABry Disease Patient-Reported Outcome-GastroIntestinal (FABPRO-GI): A new Fabry disease-specific gastrointestinal outcomes instrument

**DOI:** 10.1007/s11136-021-02847-9

**Published:** 2021-04-29

**Authors:** Alan L. Shields, Roger E. Lamoureux, Fiona Taylor, Jay A. Barth, Andrew E. Mulberg, Vivian Kessler, Nina Skuban

**Affiliations:** 1 Adelphi Values, 290 Congress Street, 6th Floor, Boston, MA 02210 USA; 2 Amicus Therapeutics, Inc., Cranbury, NJ USA

**Keywords:** Fabry disease, FABPRO-GI, Patient-reported outcomes, Gastrointestinal signs and symptoms

## Abstract

**Purpose:**

Fabry disease is a rare multisystemic disorder caused by functional deficiency of the lysosomal enzyme alpha-galactosidase A. Gastrointestinal (GI) signs and symptoms are among the earliest clinical manifestations in patients with Fabry disease but are often nonspecific, misdiagnosed, and untreated. No instruments have been developed specifically to assess GI signs and symptoms in Fabry disease. The **FAB**ry disease **P**atient-**R**eported **O**utcome-**G**astro**I**ntestinal (FABPRO-GI) was developed to address this unmet need and is intended for use in clinical trials (24-h FABPRO-GI) and real-world settings (7-day FABPRO-GI).

**Methods:**

Findings from a literature review, expert advisory meetings, and patient concept elicitation interviews (CEIs) were summarized into conceptual models. These conceptual models were used to develop preliminary versions of the 24-h and 7-day FABPRO-GI. Cognitive debriefing interviews (CDIs) were conducted with additional patients to assess content validity, including understandability, relevance, and comprehensiveness of the preliminary versions of the 24-h and 7-day FABPRO-GI.

**Results:**

Literature review (*n* = 17 articles), expert advisory meetings (*n* = 5), and patient CEIs (*n* = 17) identified mostly overlapping Fabry disease-related GI signs and symptoms, including abdominal cramps, bloating, and diarrhea, and informed development of the preliminary 24-h and 7-day FABPRO-GI. CDIs (*n* = 15) provided evidence of content validity and informed revisions of the 24-h and 7-day FABPRO-GI.

**Conclusion:**

With evidence of content validity, the 24-h and 7-day FABPRO-GI are the first Fabry disease-specific patient-reported outcomes to assess GI signs and symptoms in patients with Fabry disease with potential for use in clinical trials and real-world settings, respectively.

**Supplementary Information:**

The online version contains supplementary material available at 10.1007/s11136-021-02847-9.

## Plain English Summary

Fabry disease is a rare disorder caused by the deficiency of a specific enzyme called alpha-galactosidase A, which is required for the proper function of most cells throughout the body. Although gastrointestinal (GI) signs and symptoms are common in patients with Fabry disease and often appear early in the course of the disease, they are often misdiagnosed or untreated. No instruments have been developed to properly assess these GI signs and symptoms in patients with Fabry disease. This study attempted to develop assessment tools to effectively evaluate GI signs and symptoms in this patient population in clinical trial and real-world settings. Using information obtained from a literature search, expert advisory meetings and patient interviews, investigators were able to identify a set of GI signs and symptoms to include in a 24-h and a 7-day **FAB**ry disease **P**atient-**R**eported **O**utcome-**G**astro**I**ntestinal (FABPRO-GI) questionnaire for use in clinical trials and real-world settings, respectively. These questionnaires may aid early diagnosis and monitoring of treatment effects in Fabry disease.

## Introduction

Fabry disease is an X-linked multisystemic disorder caused by pathogenic *GLA* variants that result in functional deficiency of the lysosomal enzyme α-galactosidase A (α-Gal A) [1, 2]. Progressive accumulation of globotriaosylceramide and related glycosphingolipids can be detected in most cell types and visceral tissues [1, 2]. Glycosphingolipid accumulation can promote several pathological processes including the activation of Toll-like receptors, triggering inflammation and fibrosis cascades [3–5], and lead to a broad range of manifestations including gastrointestinal (GI) signs and symptoms, neuropathic pain, cardiomyopathy, stroke, and renal insufficiency [1, 6].

GI complaints are among the earliest and most frequent general complaints in patients with Fabry disease, affecting more than 50% of all patients with women being slightly more affected than men [7–11]. More specifically, abdominal pain was reported by 28% of adult patients and 49% of pediatric patients enrolled in the Fabry Outcome Survey [7] and by 21% of females and 13% of males enrolled in the Fabry Registry [12]. In addition, diarrhea, often associated with significant urgency and frequency [13], was reported in 19% of adult patients and 25% of pediatric patients from the Fabry Outcome Survey [7] and 19% of females and 12% of males enrolled in the Fabry Registry [12].

Despite their negative impact on the patient’s health-related quality of life, GI signs and symptoms are nonspecific and often overlooked in the management of Fabry disease [11]. In a study of 108 adult patients with Fabry disease enrolled in the Fabry Outcome Survey, health-related quality of life as assessed by EQ-5D questionnaire was significantly lower in patients with GI signs and symptoms compared with patients without GI signs and symptoms [7]. Many patients experience nonspecific Fabry disease-related GI signs and symptoms that resemble those of GI disorders and are prone to be misdiagnosed [14, 15]. For example, a study of 33 patients with Fabry disease and GI signs and symptoms showed that 16/25 adult patients and 2/8 pediatric patients exhibited GI signs and symptoms that resembled those of functional GI disorders [14].

Improvements in GI signs and symptoms have been reported in patients receiving migalastat, a small molecule pharmacological chaperone, or enzyme-replacement therapy (ERT) with recombinant human α-Gal A [20–22]. In addition, patients receiving migalastat demonstrated improvement in the only randomized controlled trial conducted with a validated GI instrument [23]. The effect of migalastat on GI signs and symptoms was assessed in ERT-naive patients from the phase 3 FACETS study using the Gastrointestinal Symptom Rating Scale (GSRS) [21, 23]. Following 6 months of migalastat treatment, a significantly greater proportion of patients receiving migalastat achieved clinically meaningful improvement in the GSRS diarrhea domain compared with placebo (*P* = 0.02) [23]. Infusion with agalsidase alfa was associated with a statistically significant reduction (14%; *P* < 0.05) from baseline in the prevalence of abdominal pain in a study of 58 patients from the Fabry Outcome Survey [7]. Similarly, postprandial pain, nausea, and vomiting substantially improved from baseline following 24 weeks of agalsidase beta treatment in an open-label study of 16 pediatric patients [24].

Although these studies provide evidence of the effect of ERT and migalastat treatment on GI signs and symptoms, none of the survey instruments used were developed for patients with Fabry disease or validated for use in this patient population, including the GSRS [21]. This observation suggests the need for a content-valid, psychometrically sound, and clinically interpretable Fabry disease-specific instrument to assess GI signs and symptoms. This report describes the development of the FABry disease Patient-Reported Outcome-Gastro Intestinal (FABPRO-GI) to assess Fabry disease-related GI signs and symptoms. Given that patient-reported outcomes (PROs) can provide value outside clinical trials for aspects of disease management such as treatment monitoring in clinical practice [25], PROs have been developed for use in multiple settings [26]. For this purpose, 2 versions of the FABPRO-GI were developed: the 24-h FABPRO-GI for clinical trials and the 7-Day FABPRO-GI for real-world settings.

## Methods

**Fig. 1:**
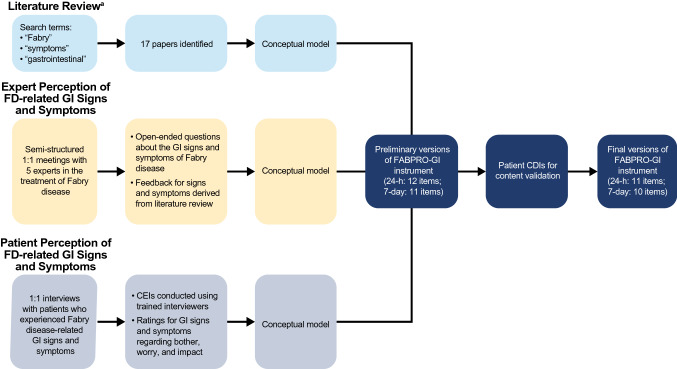
FABPRO-GI development process. *CDI* cognitive debriefing interview, *CEI* concept elicitation interviews, *FABPRO-GI* FABry disease Patient-Reported Outcome-Gastrointestinal, *GI* gastrointestinal; *h* hour. ^a^Articles identified in the literature review were considered relevant if they focused primarily on the GI symptoms of Fabry disease. Articles were excluded if they focused primarily on the pathogenesis, genetics, or molecular histology of Fabry disease; focused primarily on non-GI symptoms of Fabry disease; or discussed solely Fabry disease in a population <16 years of age

Consistent with measurement best practices [27] and regulatory guidance on the development of PROs [28], the 24-h and 7-day FABPRO-GI were developed based on results from a comprehensive literature review, expert advisory meetings, and patient concept elicitation interviews (CEIs) (Fig. 1).

### Literature Review and Expert Advisory Meetings

MEDLINE, Embase, and PsycINFO were searched using the OvidSP platform for the time period 2005-July 1, 2015 to identify peer-reviewed articles relating to GI signs and symptoms of Fabry disease. Search terms were “Fabry,” “symptoms,” and “gastrointestinal.” Abstracts were screened to identify relevant articles, and additional references were identified by manually inspecting the reference lists of relevant articles from the search as well as targeted searches of the National Fabry Disease Foundation (NFDF) patient advocacy website (https://www.fabrydisease.org/). Relevant articles primarily focusing on the GI signs and symptoms of Fabry disease were identified, and articles were excluded if they primarily focused on the pathogenesis, genetics, or molecular histology of Fabry disease; primarily focused on non-GI signs and symptoms of Fabry disease; or solely discussed Fabry disease in a population aged <16 years.

Semi-structured 1:1 meetings with experts experienced in the treatment of Fabry disease were conducted via telephone by trained interviewers between December 2015 and April 2016. Meetings were designed to give the experts an opportunity to spontaneously describe GI sign- and symptom-level concepts by asking them open-ended questions about the GI signs and symptoms of Fabry disease. In addition, descriptive data were collected relating to the geographic location, years of experience treating patients with Fabry disease, medical specialty, approximate number of patients with Fabry disease seen in a typical month, and work setting.

### Patient Perspective

#### Patients

One-on-one interviews were conducted with patients with Fabry disease. Patients were recruited between September and November 2015 through the NFDF email list. Eligible patients were fluent in English, aged ≥16 years, and self-reported having received a diagnosis of Fabry disease and having ≥1 GI sign or symptom (eg, diarrhea, constipation, abdominal pain) in the 14 days prior to study entry.

#### Patient interviews

Semi-structured 1:1 CEIs with patients with Fabry disease were conducted in person or via telephone by trained interviewers. Interviewers recorded patient expressions of Fabry disease-related GI signs or symptoms including overall impression, severity, occurrence, frequency, duration, and changes over time. Patients were asked to rate each current GI sign or symptom with respect to the level of bother, worry, and impact it caused on a scale of 0 (no bother/worry/impact) to 10 (most bothersome/worrisome/impactful). Patients were also asked to report the 5 signs or symptoms they would most like to see improve if an effective treatment was available to them.

#### Development of Conceptual Models

Concepts were organized into and presented in conceptual models defined as heuristic classification schemes linking a disease state to its proximal and increasingly distal health outcomes [29]. Conceptual models were developed separately based on Fabry disease-related GI signs and symptoms from the literature review, expert advisory meetings, and CEIs (patient interviews) (Fig. 1). Experts were asked to review and provide feedback on the Fabry disease-related GI sign and symptom conceptual model derived from the literature review.

#### Development of the 24-h and 7-day FABPRO-GI

The preliminary versions of the 24-h and 7-day FABPRO-GI were drafted based on results consolidated from conceptual models for the literature review, expert advisory meetings, and patient CEIs according to measurement best practices [27] and regulatory guidance [28] (Fig. 1). Items assessing signs or symptoms asked patients to rate the severity of the specified sign or symptom “at its worst” in the 24 h (24-h FABPRO-GI) or 7 days (7-day FABPRO-GI) prior to assessment on an 11-point (0–10) numeric rating scale. The preliminary versions of the 24-h and 7-day FABPRO-GI were then subject to cognitive debriefing interviews (CDIs) for readability and patient comprehension, and results informed the final versions (Fig. 1).

Patients (*n* = 15) participating in CDIs were recruited in September 2016 through the NFDF and met the same eligibility criteria as patients participating in the CEIs. CDIs were conducted in person by a trained interviewer using a semi-structured CDI guide and consisted of 2 stages: a “think-aloud” stage in which the patient completed the questionnaire while verbalizing the process (without any input from the interviewer) and a discussion stage, in which patients reported whether the instructions, items, response options, and recall periods were relevant and appropriate for the questionnaires. Patients were also asked if they would suggest the questionnaires be revised to make them more relevant, comprehensive, and interpretable. CDIs were then transcribed, coded, and analyzed to determine whether revisions to the 24-h and 7-day FABPRO-GI were necessary.

#### Data Handling and Analysis

Transcribed meetings and interviews were anonymized, coded, and analyzed using descriptive statistics and qualitative analytic methods. The transcripts were entered into ATLAS.ti Version 7.5.6 (ATLAS.ti Scientific Software Development GmbH; Berlin, Germany) and coded by researchers by linking transcript text to a code from the codebook that best characterized the concept. For CEIs, each code contained information about the domain (eg, symptom), root concept (eg, abdominal pain), and descriptor (eg, severity, frequency, duration, or location). For CDIs, each code contained information about interpretation of questionnaire instructions, items, and response options, experience of the symptom being assessed, and suggestions for revision. Development of the codebook, a comprehensive list of all codes used to characterize important segments of transcript text, was an iterative process in which codes were added, deleted, or merged as each interview transcript became available. For CEIs, saturation, the point at which no new information is gained from additional interviewees, was evaluated in a stepwise process.

The frequency of experts who indicated that a given sign or symptom concept was part of the Fabry disease-related GI experience for patients was determined by counting the number of unique experts who mentioned the concept at least once during the expert advisory meeting. The frequency of experts who considered each concept to be an important treatment outcome was also determined. Furthermore, concept frequency was determined for patients with Fabry disease, as well as a qualitative analysis of bother, worry, and impact rating data. Data characterizing the study population of experts and patients with Fabry disease were summarized descriptively.

## Results

### Literature review

Seventeen relevant articles were identified from the literature search (Online Resource Figure S1), including 9 primary research articles, 7 review articles, and 1 resource document from the NFDF website. Review of these sources identified 14 Fabry-related GI signs and symptoms (in decreasing order of frequency): diarrhea, abdominal pain, nausea, vomiting, bloating, constipation, weight loss, abdominal cramping, chest pain (non-cardiac, presumed esophageal origin), excessive belching, feeling of satiety, gas, indigestion, and reflux. Concepts relating to GI signs and symptoms of Fabry disease were subsequently organized into a literature-based conceptual model.

### Expert Perspectives

**Fig. 2:**
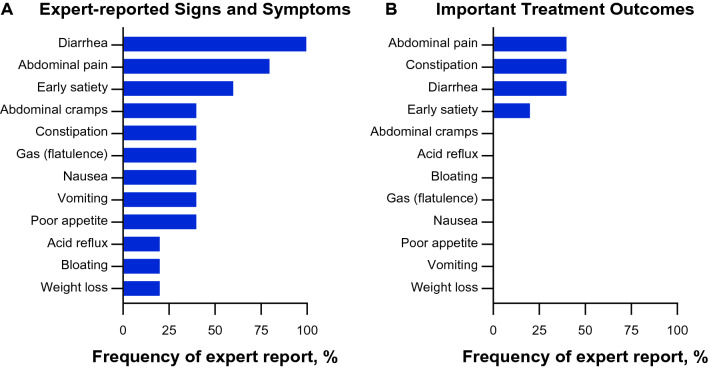
Expert-reported (A) GI signs and symptoms and (B) important treatment outcomes in Fabry disease. *GI* gastrointestinal

Five experts from the United States (*n* = 3) and England (*n* = 2) participated in advisory meetings. These individuals worked in a variety of clinical settings, had treated patients with Fabry disease for between 15 and 24 years, and reported seeing approximately 6–80 patients with Fabry disease per month. Together, these experts reported a total of 12 GI signs and symptoms of Fabry disease: diarrhea, abdominal pain, early satiety, abdominal cramps, constipation, gas (flatulence), nausea, vomiting, poor appetite, acid reflux, bloating, and weight loss (Fig. 2A). The 3 signs and symptoms reported by most experts (diarrhea [*n* = 5; 100%]; abdominal pain [*n* = 4; 80%]; and early satiety [*n* = 3; 80%]) were also identified by these experts as important treatment outcomes from their perspective and/or the patients’ perspective (*n* = 2; *n* = 2; and *n* = 1, respectively) (Fig. 2B). Other treatment outcomes deemed as important for patients with Fabry disease by the experts were related to constipation (*n* = 2; 40%), asymptomatic status (*n* = 1; 20%), and less GI sign or symptom interference in the patient’s life (*n* = 1; 20%). Upon reviewing the literature-based conceptual model, 1 expert agreed with all GI signs and symptoms in the model and 3 (60%) experts suggested removing ≥1 sign or symptom (reflux [*n* = 2]; weight loss [*n* = 2]; chest pain [*n =* 1]; indigestion [*n* = 1]).

Four experts believed GI signs and symptoms of Fabry disease varied with patient age, and all experts agreed that the presentation of GI signs and symptoms in Fabry disease varied with patient sex. Regarding treatment of these signs and symptoms, experts confirmed that no Fabry disease-specific treatment was available to them to prescribe to their patients and, instead, they relied upon treatments used for other GI signs or symptoms including medications such as antacids, antispasmodic drugs, loperamide, and metoclopramide, as well as dietary changes.

### Patient Perspectives

Nineteen patients were interviewed and 17 were included in the analysis (2 patients were excluded based on a diagnosis of gastroparesis reported during the CEI; Table 1). Among patients contributing to the analysis, 10 patients (58.8%) were female, the mean (range) age was 33.7 (16.7–60.8) years, and 12 patients (70.6%) were currently receiving ERT. Most patients self-reported the severity of their Fabry disease-related GI signs and symptoms as moderate (*n* = 11; 64.7%) or mild (*n* = 3; 17.6%). To determine whether an adequate sample size had been achieved for identifying GI signs and symptoms, a concept saturation grid was developed, which showed elicitation of 92.3% of all concepts in the first 75% of interviews, supporting the achievement of saturation (Online Resource Table S1).

**Table 1:** Demographics and disease characteristics of patients with Fabry disease participating in CEIs and CDIs

	CEIs (*N* = 17)	CDIs (*N* = 15)
Age, years, mean (SD)	33.7 (14.2)	39.6 (16.7)
Female, n (%)	10 (58.8)	11 (73.3)
Race, n (%)		
White/Caucasian	11 (64.7)	13 (86.7)
Black or African American	–	2 (13.3)
Other	5 (29.4)	
Not answered	1 (5.9)	
Highest level of education, n (%)		
High school diploma (or GED) or less	6 (35.3)	7 (46.7)
Some college or certificate program	4 (23.5)	3 (20.0)
College or university degree (2- or 4-year)	5 (29.4)	3 (20.0)
Graduate degree	1 (5.9)	2 (13.3)
Other	1 (5.9)^a^	–
Time since diagnosis, years, mean (SD)	11.8 (12.2)	7.9 (6.2)
Health status, n (%)^b^		
Excellent	1 (5.9)	–
Very good	6 (35.3)	2 (13.3)
Good	6 (35.3)	9 (60.0)
Fair	3 (17.6)	4 (26.7)
Poor	1 (5.9)	–
Fabry disease-related GI symptom severity, n (%)^b^		
Very mild	1 (5.9)	1 (6.7)
Mild	3 (17.6)	2 (13.3)
Moderate	11 (64.7)	11 (73.3)
Severe	1 (5.9)	1 (6.7)
Very severe	1 (5.9)	–
Current medications, n (%)^b,c,d^		
Prophylactic pain agents^e^	6 (35.3)	4 (26.7)
NSAIDs	5 (29.4)	3 (20.0)
Analgesics^f^	5 (29.4)	5 (33.3)
Anti-nausea agents	3 (17.6)	3 (20.0)
Antacids	3 (17.6)	4 (26.7)
Docusate	2 (11.8)	2 (13.3)
Enemas	2 (11.8)	—
Prokinetics	2 (11.8)	3 (20.0)
Anti-diarrheal agents	1 (5.9)	1 (6.7)
Bismuth	1 (5.9)	2 (13.3)
GI disturbance medication	1 (5.9)	4 (26.7)
Anti-spasmodic agents	–	1 (6.7)
Other	2 (11.8)	5 (33.3)
Abdominal surgeries, n (%)^b,c^		
Hysterectomy, n (%)	3 (17.6)	3 (20.0)
Removal of appendix	2 (11.8)	3 (20.0)
Removal of gallbladder	1 (5.9)	4 (26.7)

*CDI* cognitive debriefing interview, *CEI* concept elicitation interview, *GED* general educational development, *GI* gastrointestinal, *NSAID* nonsteroidal anti-inflammatory drugs, *SD* standard deviation

^a^Patients still attending school

^b^Patient-reported

^c^Not mutually exclusive

^d^Enzyme replacement therapy was excluded

^e^For example, phenytoin, carbamazepine, or gabapentin

^f^For example, narcotics, opioids such as codeine, hydrocodone, Demerol, OxyContin, or Percocet

**Fig. 3:**
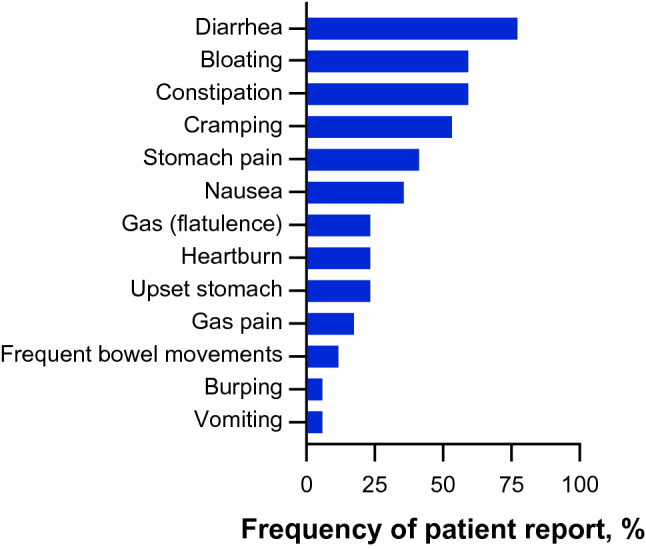
Patient-reported GI signs and symptoms in Fabry disease. *GI* gastrointestinal

Of the Fabry disease-related GI signs and symptoms mentioned by patients during the CEIs, the most commonly reported (by ≥ 50% of patients) were diarrhea (by *n* = 13; 76.5%), bloating (*n* = 10; 58.8%), constipation (*n* = 10; 58.8%), and cramping (*n* = 9; 52.9%) (Fig. 3). For the signs and symptoms reported by ≥50% of patients, the reported sign or symptom severity ranged from mild (*n* = 1 of 7) to severe (*n* = 4 of 7) for diarrhea, and most patients reported their bloating as mild to moderate (*n* = 7 of 8), their constipation as severe (*n* = 6 of 9), and their cramping as “intense,” “severe,” or “very painful” (*n* = 6 of 8).

Patient perception of the bother, worry, and impact of each GI sign or symptom was rated on a scale of 0 to 10, where higher scores indicate more bother, worry, or impact, and is reported in Table 2. Among the GI signs and symptoms reported by ≥50% of patients (diarrhea, bloating, constipation, and cramping), mean bother ratings ranged from 4.9 to 6.5, mean worry ratings ranged from 3.5 to 5.5, and mean impact ratings ranged from 3.3 to 6.0. Diarrhea was the symptom most frequently ranked as the most important to improve (*n* = 6) and was most frequently ranked among the top 5 signs and symptoms (*n* = 11), followed by bloating (*n* = 9) and constipation (*n* = 8). The 13 Fabry disease-related GI sign and symptom concepts reported by patients during the CEIs were organized into a patient-centric conceptual model.

**Table 2:** Patient-reported GI signs and symptoms and their bother, worry, and impact

Concept^a^	Ratings/Patients^b^	Bother rating^c^		Worry rating^c^		Impact rating^c^	
		*n*	Mean (SD)	*n*	Mean (SD)	n	Mean (SD)
Diarrhea (*n* = 13)	11/13	11	6.4 (2.0)	11	3.5 (2.9)	11	5.1 (3.3)
Bloating (*n* = 10)	9/10	9	4.9 (2.3)	9	3.6 (3.2)	9	3.3 (3.3)
Constipation (*n* = 10)	9/10	9	6.4 (3.0)	9	5.5 (3.3)	9	5.8 (2.8)
Cramping (*n* = 9)	8/9	8	6.5 (2.3)	8	4.4 (2.7)	7	6.0 (3.4)
Stomach pain (*n* = 7)	7/7	7	6.1 (2.9)	7	4.9 (1.7)	7	5.0 (2.9)
Nausea (*n* = 6)	5/6	5	6.2 (2.8)	5	2.4 (1.9)	5	4.0 (3.5)
Gas (*n* = 4)	4/4	4	3.8 (3.3)	4	4.5 (5.3)	4	5.0 (4.2)
Heartburn (*n* = 4)	4/4	4	5.5 (3.7)	4	4.5 (3.8)	4	3.8 (1.7)
Upset stomach (*n* = 4)	3/4	3	5.7 (2.1)	3	3.0 (2.6)	3	5.0 (4.4)
Gas pain (*n* = 3)	2/3	2	6.5 (0.7)	2	3.0 (4.2)	2	4.5 (4.9)
Frequent bowel movements (*n* = 2)	1/2	1	7.0	1	8.0	1	6.0
Burping (*n* = 1)	0/1	0	–	0	–	0	–
Vomiting (*n* = 1)	1/1	1	8.0	1	4.0	1	5.0

*CEI* concept elicitation interview, *GI* gastrointestinal, *SD* standard deviation

^a^Based on the CEIs

^b^Indicates the number of patients who provided a symptom rating out of the total number of patients reported experiencing the symptom during the open-ended discussion. The number of patients who had the opportunity to provide a rating for the symptom may be less than the total frequency count for some symptoms as patients were only able to rate symptoms that the interviewer specifically asked about during the interview, prior to the complete analysis of qualitative data

^c^Rated on a 0–10 scale where a higher score indicates more bother, worry, or impact

### Development of the 24-h and 7-day FABPRO-GI

**Fig. 4:**
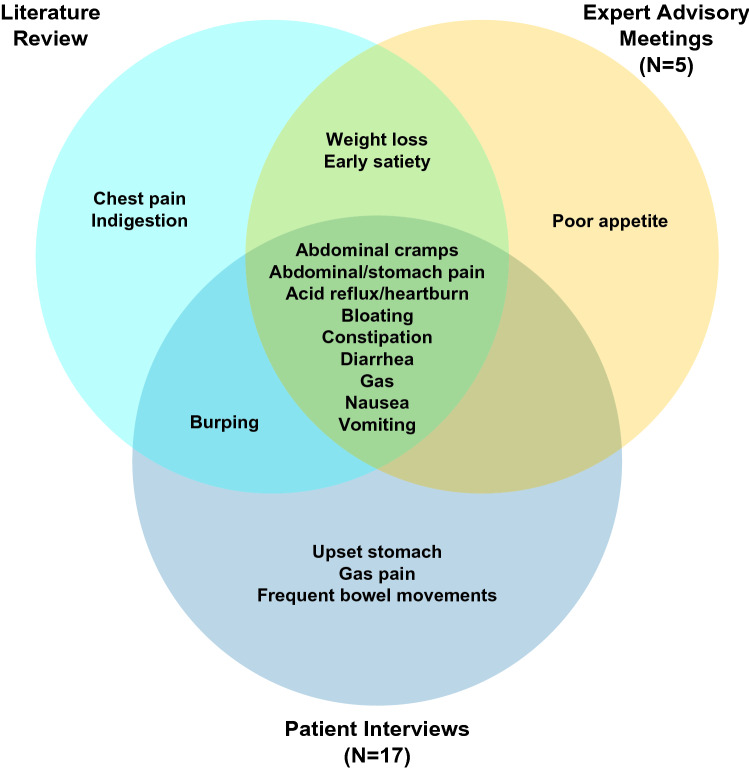
Fabry disease-related GI sign and symptom concepts as reported in the literature, by experts, and by patients. *GI* gastrointestinal

Three conceptual models were derived from the literature review, expert advisory meetings, and patient CEIs, which showed largely overlapping GI signs and symptoms (Fig. 4). The signs and symptoms derived from the conceptual models informed the target concepts of interest (bloating; stomach pain; cramping; nausea; acid reflux; heartburn; indigestion; constipation; severity, frequency, and consistency of diarrhea; and bowel movement frequency), and 2 preliminary FABPRO-GI questionnaires were developed to assess those concepts (1) in the 24 h prior to assessment during clinical trials (Table 3) and (2) in the 7 days prior to assessment in real-world settings (Table 4). The concepts assessed by the 7-day FABPRO-GI are the same as those assessed by the 24-h FABPRO-GI, with the difference that the 7-day FABPRO-GI uses a recall period of “the past 7 days” and asks subjects to report the average severity of symptoms over that period.

**Table 3:** Items on the Preliminary 24-h FABPRO-GI^a^

Domain	Item
Disease-related GI sign and symptom severity	1. Over the past 24 h, how severe was your **worst bloating**?
	2. Over the past 24 h, how severe was your **worst stomach pain**?
	3. Over the past 24 h, how severe was your **worst cramping**?
	4. Over the past 24 h, how severe was your **worst nausea**?
	5. Over the past 24 h, how severe was your **worst acid reflux**?
	6. Over the past 24 h, how severe was your **worst heartburn**?
	7. Over the past 24 h, how severe was your **worst indigestion**?^b^
	8. Over the past 24 h, how severe was your **worst constipation**?
	12. Over the past 24 h, how severe was your **worst diarrhea**?
Frequency of bowel movements	9. Over the past 24 h, how many **bowel movements** did you have?
Frequency of diarrhea	10. Over the past 24 h, how many times did you have **diarrhea**?
Consistency of diarrhea	11. Over the past 24 h, what was the consistency of your **worst diarrhea**?

The bolded text is as it appears on the questionnaire to emphasize the rating is for “worst” (24 h FABPRO-GI) and “average” (7-day FABPRO-GI) symptoms, respectively

*FABPRO-GI* FABry Disease Patient Reported Outcome-Gastrointestinal

^a^Items were scored on a 0–10 scale, where “0” indicated the absence of the sign or symptom and “10” indicated worst possible

^b^Item 7 was removed from the final 24-h FABPRO-GI

**Table 4:** Items on the Preliminary 7-day FABPRO-GI^a^

Domain	Item
Disease-related GI sign and symptom severity	1. Over the past 7 days, how severe was your **bloating on average**?
	2. Over the past 7 days, how severe was your **stomach pain on average**?
	3. Over the past 7 days, how severe was your **cramping on average**?
	4. Over the past 7 days, how severe was your **nausea on average**?
	5. Over the past 7 days, how severe was your **acid reflux on average**?
	6. Over the past 7 days, how severe was your **heartburn on average**?
	7. Over the past 7 days, how severe was your **indigestion** on average?^b^
	8. Over the past 7 days, how severe was your **constipation on average**?
	11. Over the past 7 days, how severe was your **diarrhea on average**?
Frequency of bowel movements	9. Over the past 7 days, how many **bowel movements per day** did you have **on average**?
Frequency of diarrhea	10. Over the past 7 days, how many times **per day** did you have **diarrhea on average**?

The bolded text is as it appears on the questionnaire to emphasize the rating is for “worst” (24 h FABPRO-GI) and “average” (7-day FABPRO-GI) symptoms, respectively

*FABPRO-GI* FABry Disease Patient Reported Outcome-Gastrointestinal

^a^Items were scored on a 0–10 scale, where “0” indicated the absence of the sign or symptom and “10” indicated worst possible

^b^Item 7 was removed from the final 7-day FABPRO-GI

To evaluate the preliminary content of the 24-h and 7-day FABPRO-GI, 15 patients (Table 1) with Fabry disease participated in CDIs to provide feedback on all instructions, items, and response options of the 24-h FABPRO-GI, and the instructions and Items 2 (stomach pain), 8 (constipation), and 11 (diarrhea severity) of the 7-day FABPRO-GI. Eleven patients (73.3%) were female, the mean (range) age was 39.6 (16.9–64.0) years, and the majority (*n =* 11; 73.3%) reported their Fabry disease-related GI sign and symptom severity as moderate (Table 1).

For the 24-h FABPRO-GI questionnaire, all but 1 patient who provided an interpretable response (*n* = 13; 92.9%) interpreted the instructions as intended. The patient who did not interpret a portion of the instructions as intended showed some confusion at first but proceeded to answer items as intended. In addition, most patients (*n* = 14; 93.3%) understood the 24-h recall period within the instructions. All 13 patients who provided an interpretable response interpreted the instructions of the 7-day FABPRO-GI as intended, and all patients (*n* = 15) understood the 7-day recall period.

Overall, patients interpreted all items of the 24-h and 7-day FABPRO-GI questionnaires as intended. It is notable that 2 patients (13.3%) did not interpret Item 7 (indigestion) of the 24-h FABPRO-GI as intended, and even those who interpreted Item 7 as intended were thinking about a wide variety of different symptoms, many of which overlapped with concepts already in the questionnaire. For Items 1–10 and 12, at least 92.3% of patients reported experiencing the sign or symptom either within or prior to the 24-h recall period used in the questionnaire, whereas Item 11 (diarrhea consistency) applied only to 4 patients (26.7%) as they were the only patients who reported ≥1 episode of diarrhea within the past 24 h. All patients interpreted the response scales of the 24-h FABPRO-GI as intended by the developers. In addition, all patients stated the questionnaire was easy to complete. Two patients (14.3%) indicated 1 or more items were repetitive, 4 patients (26.7%) suggested deleting one or more items, and the items most frequently ranked as the most important were Items 8 (constipation, *n* = 8; 57.1%), Items 10, 11, and 12 (diarrhea frequency/consistency/severity, *n* = 8; 57.1%), and Item 2 (stomach pain, *n* = 2; 42.9%).

For the 7-day FABPRO-GI, 75% (*n* = 9 of 12), 90.9% (*n* = 8 of 11), and 90.9% (*n* = 8 of 11) of respondents interpreted Item 2 (stomach pain), Item 8 (constipation), and Item 11 (diarrhea) as intended, respectively. Two patients (16.7%) assessed severity of their stomach pain based on frequency of occurrence rather than severity. One patient misinterpreted the recall period for each of the 3 items.

On the basis of patient feedback, revisions to the 24-h FABPRO-GI questionnaire included removing Item 7 (indigestion) and the addition of a skip pattern for Items 11 (diarrhea consistency) and 12 (diarrhea severity) for patients who had not experienced diarrhea in the past 24 h. The final 24-h FABPRO-GI consisted of 11 items. Item 7 was also removed from the 7-day FABPRO-GI based on the feedback received to produce the final 10-item questionnaire.

## Discussion

The results of this study provide evidence of the content validity of the 24-h and 7-day FABPRO-GI in assessing concepts that are relevant to Fabry disease and important to affected patients and that they can do so in ways that patients can easily understand and complete. Informed by the literature, expert advice, and patient input, the 24-h and 7-day FABPRO-GI can be used to assess GI signs and symptoms in patients with Fabry disease. The general understandability, relevance, and comprehensiveness of the 24-h and 7-day FABPRO-GI were evaluated and confirmed in a group of patients aged ≥16 years with Fabry disease with minor modifications to the questionnaires. These were developed in accordance with regulatory guidance [28] and have the potential for use in real-world settings to increase understanding of the GI signs and symptoms experienced by patients with Fabry disease, and in clinical trials to assess the effects of therapy on Fabry disease-related GI signs and symptoms. Moreover, patients with a variety of genotypes were included in this study to represent the broad phenotypic spectrum of Fabry disease.

GI signs and symptoms, often the first clinical manifestation of Fabry disease, are nonspecific, likely contribute to long diagnostic delays and misdiagnoses experienced by patients, and impose substantial psychological stress on patients [11]. During interviews for this study, patients reported experiencing delays and difficulties in obtaining proper diagnosis and treatment. Numerous possible differential diagnoses exist for nonspecific GI signs and symptoms. For example, diarrhea episodes alternating with normal bowel activity or constipation, which may occur in a patient with Fabry disease, may alternatively indicate diarrhea-predominant irritable bowel syndrome [11]. Patients with Fabry disease initially presenting with abdominal pain have received wide-ranging misdiagnoses including non-specific pain, food-borne illness, dyspepsia, and gastroesophageal reflux and received treatment for these disorders prior to receiving the correct diagnosis and treatment [15]. Moreover, GI signs and symptoms are associated with psychological stress that may exacerbate pain and discomfort in patients with Fabry disease [11].

To date, no instruments are in clinical use to evaluate Fabry disease-related GI signs and symptoms. Given the nonspecificity of GI signs and symptoms associated with Fabry disease, this may hinder timely diagnosis and highlights the importance of developing a Fabry disease-specific instrument for assessing GI signs and symptoms.

The 24-h and 7-day FABPRO-GI are the first Fabry disease-specific PROs to assess GI signs and symptoms in patients with Fabry disease. Gastrointestinal signs and symptoms in patients with Fabry disease were previously evaluated via patient interviews and instruments designed to assess other gastrointestinal disorders including the GSRS [30, 23] and Rome III criteria [31, 14] (now Rome IV [32]). Given the paucity of PROs developed for Fabry disease, the 24-h and 7-day FABPRO-GI may provide new insights into Fabry disease-related GI signs and symptoms that may facilitate their recognition in patients with Fabry disease participating in clinical trials and those in real-world settings.

The methodology used to develop the 24-h and 7-day FABPRO-GI is similar to those of other recently developed Fabry disease-specific PROs. A self-reported questionnaire to evaluate patient treatment expectations for Fabry disease regarding aspects such as long-term efficacy, impact on daily living, and effective treatment of signs and symptoms was recently developed based on a review of the literature and patient interviews [33]. As content validity and test and retest reliability were demonstrated, this questionnaire was considered to be a suitable instrument for assessing patients with Fabry disease and is currently being used in a phase 4 study [33]. Similarly, a PRO for the evaluation of neuropathic pain in patients with Fabry disease was developed based on CEIs and CDIs for use in pivotal clinical trials [34].

There were several limitations to this study. One is the small number of patients who participated in CEIs (*n* = 17) and CDIs (*n* = 15). However, at least one meta-analytic inquiry across 26 CE studies indicates that small sample sizes have proven adequate for these types of specific inquiries [35]. The consistency of the present saturation results with the referenced meta-analysis enhances confidence in the reliability of conclusions drawn from the research presented here. Second, although this study describes the development and content validation of the 24-h and 7-day FABPRO-GI, evaluation of psychometric performance and score interpretation of these outcome measures in clinical studies is pending.

## Conclusions

The 24-h and 7-day FABPRO-GI can potentially be used in clinical trials and real-world settings, respectively, to monitor and help bring attention to often underrecognized and untreated GI signs and symptoms in patients with Fabry disease.

## Supplementary Information

Below is the link to the electronic supplementary material.

Supplementary file1 (PDF 329 KB)

## Data Availability

All data relevant to this study are included in the article or were uploaded as supplementary material.

## Abbreviations

α-Gal A: α-Galactosidase A
CDI: Cognitive debriefing interviews
CEI: Concept elicitation interviews
ERT: Enzyme replacement therapy
FABPRO-GI: FABry Disease Patient-Reported Outcome-GastroIntestinal
GI: Gastrointestinal
GSRS: Gastrointestinal Symptom Rating Scale
NFDD: National Fabry Disease Foundation
PROs: Patient-reported outcomes
