# Supplementary material for: FABry Disease Patient-Reported Outcome-GastroIntestinal (FABPRO-GI): A new Fabry disease-specific gastrointestinal outcomes instrument
Source: Qual Life Res. 2021 Apr 29;30(10):2983–94. doi: 10.1007/s11136-021-02847-9 (PMC8481178; doi:10.1007/s11136-021-02847-9)
Supplement: Supplementary file 1 — Supplementary file1 (PDF 329 KB) [file 11136_2021_2847_MOESM1_ESM.pdf]

SUPPLEMENTARY TABLES AND FIGURES

FABry Disease Patient-Reported Outcome-GastroIntestinal (FABPRO-GI): A New Fabry Disease-specific Gastrointestinal Outcomes Instrument

Journal: *Quality of Life Research*

Alan L. Shields,[1] Roger E. Lamoureux,[1] Fiona Taylor,[1] Jay Barth,[2] Andrew E. Mulberg,[2] Vivian Kessler,[2] Nina Skuban[2]

[1]Adelphi Values, Boston, MA, USA; [2]Amicus Therapeutics, Inc., Cranbury, NJ, USA

Corresponding Author: Alan L. Shields

Adelphi Values

290 Congress Street, 6th Floor

Boston, MA 02210

Email: Alan.Shields@adelphivalues.com

Online Resource Table 1. Saturation Grid for Fabry Disease-related GI Signs and Symptoms

**Concept**	**First ~25% of interviews vs next ~25% of interviews**	**First ~50% of interviews vs next ~25% of interviews**	**First ~75% of interviews vs next ~25% of interviews**	**Total**	**Number of concepts elicited in first 75% of interviews/ Total concepts elicited (%)**
Diarrhea	5 vs 3	8 vs 1	9 vs 4	13	12/13 (92.3%)
Bloating	4 vs 1	5 vs 3	8 vs 2	10	
Constipation	4 vs 3	7 vs 3	10 vs 0	10	
Cramping	1 vs 3	4 vs 2	6 vs 3	9	
Stomach pain	2 vs 3	5 vs 2	7 vs 0	7	
Nausea	0 vs 2	2 vs 2	4 vs 2	6	
Gas	2 vs 1	3 vs 1	4 vs 0	4	
Heartburn	1 vs 1	2 vs 2	4 vs 0	4	
Upset stomach	3 vs 0	3 vs 1	4 vs 0	4	
Gas pain	2 vs 0	2 vs 1	3 vs 0	3	
Frequent bowel movements	1 vs 1	2 vs 0	2 vs 0	2	
Burping	0 vs 0	0 vs 1	1 vs 0	1	
Vomiting	0 vs 0	0 vs 0	0 vs 1	1	

GI, gastrointestinal.

Online Resource Figure 1. Literature Search Flow Diagram[a]

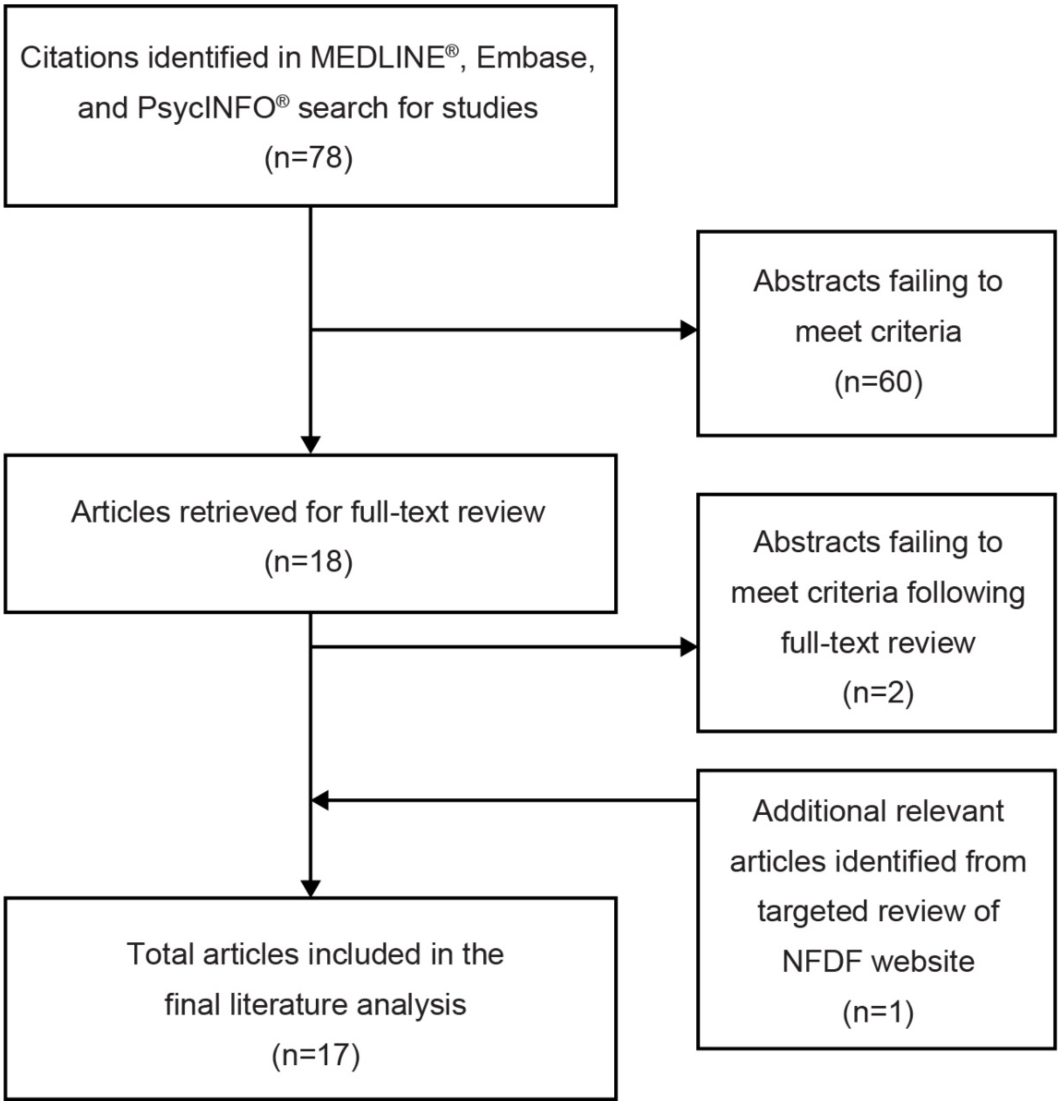

GI, gastrointestinal; NFDF, National Fabry Disease Foundation.

[a]Articles identified in the literature review were considered relevant if they primarily focused on the GI symptoms of Fabry disease. Articles were excluded if they primarily focused on the pathogenesis, genetics, or molecular histology of Fabry disease; primarily focused on non-GI symptoms of Fabry disease; or solely discussed Fabry disease in a population <16 years of age.
